# Paracetamol plus ibuprofen for the treatment of fever in children (PITCH): randomised controlled trial

**DOI:** 10.1136/bmj.a1302

**Published:** 2008-09-02

**Authors:** Alastair D Hay, Céire Costelloe, Niamh M Redmond, Alan A Montgomery, Margaret Fletcher, Sandra Hollinghurst, Tim J Peters

**Affiliations:** 1 Academic Unit of Primary Health Care, NIHR National School for Primary Care Research, Department of Community Based Medicine, University of Bristol, Bristol BS8 2AA; 2 Faculty of Health and Social Care, University of West England, Bristol

## Abstract

**Objective** To investigate whether paracetamol (acetaminophen) plus ibuprofen are superior to either drug alone for increasing time without fever and the relief of fever associated discomfort in febrile children managed at home.

**Design** Individually randomised, blinded, three arm trial.

**Setting** Primary care and households in England.

**Participants** Children aged between 6 months and 6 years with axillary temperatures of at least 37.8°C and up to 41.0°C.

**Intervention** Advice on physical measures to reduce temperature and the provision of, and advice to give, paracetamol plus ibuprofen, paracetamol alone, or ibuprofen alone.

**Main outcome measures** Primary outcomes were the time without fever (<37.2°C) in the first four hours after the first dose was given and the proportion of children reported as being normal on the discomfort scale at 48 hours. Secondary outcomes were time to first occurrence of normal temperature (fever clearance), time without fever over 24 hours, fever associated symptoms, and adverse effects.

**Results** On an intention to treat basis, paracetamol plus ibuprofen were superior to paracetamol for less time with fever in the first four hours (adjusted difference 55 minutes, 95% confidence interval 33 to 77; P<0.001) and may have been as good as ibuprofen (16 minutes, −7 to 39; P=0.2). For less time with fever over 24 hours, paracetamol plus ibuprofen were superior to paracetamol (4.4 hours, 2.4 to 6.3; P<0.001) and to ibuprofen (2.5 hours, 0.6 to 4.4; P=0.008). Combined therapy cleared fever 23 minutes (2 to 45; P=0.025) faster than paracetamol alone but no faster than ibuprofen alone (−3 minutes, 18 to −24; P=0.8). No benefit was found for discomfort or other symptoms, although power was low for these outcomes. Adverse effects did not differ between groups.

**Conclusion** Parents, nurses, pharmacists, and doctors wanting to use medicines to supplement physical measures to maximise the time that children spend without fever should use ibuprofen first and consider the relative benefits and risks of using paracetamol plus ibuprofen over 24 hours.

**Trial registration** Current Controlled Trials ISRCTN26362730.

## Introduction

Fever is a normal part of childhood illness, affecting around 70% of preschool children yearly.^1^ It can be miserable for the child, cause anxiety for parents,^2^ and be expensive for health services. Up to 40% of preschool children see a health professional for a febrile illness each year.^1^ Although fever is considered by many to be an advantageous evolutionary byproduct of the host response to infection, and as such should not be treated,^3^ the use of antipyretics is widespread. The reasons for treating fever are contested and not necessarily evidence based but include minimising discomfort, controlling the fever, and preventing febrile convulsions.

Options for treating fever include physical measures (taking cool fluids and dressing lightly) and the antipyretic drugs paracetamol (acetaminophen) and ibuprofen. Evidence for physical measures is now redundant as it mostly pertains to tepid sponging,^4^ which is no longer recommended.^5^ Paracetamol and ibuprofen have both been shown to be superior to placebo^6^ ^7^ ^8^ and ibuprofen superior to paracetamol^9^ for the relief of fever. Given that the drugs have different mechanisms of action^10^ it is possible that they are more effective together than when used alone, but the evidence to date is sparse and conflicting. Five published trials^11^ ^12^ ^13^ ^14^ ^15^ mostly tested the effects of single doses at selected time points (which can arbitrarily advantage one drug because of the difference in times to maximum effect^16^), were largely done in secondary care, and reached conflicting conclusions. Recently published UK guidelines^5^ advise the use of either drug (no preference stated) for children with fever who are unwell or distressed and state that owing to the lack of evidence the drugs should not be used together or alternately.

We carried out a community based, three arm, blinded, randomised controlled trial to investigate the relative clinical effectiveness of multiple doses (as used for most episodes of fever) of paracetamol plus ibuprofen compared with either drug alone. Our investigation into the relative cost effectiveness is reported in an accompanying paper.^17^

## Methods

We recruited and followed up children between January 2005 and May 2007 using three strategies: local, remote, and community. We invited all NHS organisations providing primary care services in Bristol to assist with recruitment to the trial, including NHS Direct, the walk-in centres, all general practices, the general practitioner out of hours cooperatives, and the emergency department of the Bristol Royal Hospital for Children.

During local recruitment the NHS sites invited parents of appropriately aged children to discuss the study with our research nurses, who were present in the waiting rooms. In the remote strategy, clinicians faxed the details of potentially eligible children to the trial administrator, who notified the research nurses. In the community strategy, parents were invited to contact the trial directly by telephone. The telephone number was promoted during local and remote recruitment and in local newspaper and radio advertisements. When parents made contact, the trial administrator notified the research nurses of potentially eligible children.

Once aware of potentially eligible children identified through any of the recruitment strategies, research nurses contacted parents by telephone to arrange a meeting (usually at home) to explain the trial fully and to verify eligibility.

### Participants

We included children if they were aged between 6 months and 6 years and were unwell with a temperature of at least 37.8°C and up to 41.0°C as a result of illnesses that could be managed at home. We excluded children if they required hospital admission; were clinically dehydrated; had recently participated in another trial; had previously participated in PITCH; had a known intolerance, allergy, or contraindication to a trial drug^18^; had a chronic neurological, cardiac, pulmonary (except asthma), liver, or renal disease; or had parents who could not read or write in English. We followed up children at 24 and 48 hours and at day 5.

### Randomisation

After written informed consent had been obtained and the baseline questionnaire completed, the research nurse telephoned a remote, automated randomisation service. Allocation to one of three trial arms (paracetamol plus ibuprofen, paracetamol alone, ibuprofen alone) was minimised^19^ by age (6-17 months *v* 18-71 months), severity of fever (37.8°C to 38.9°C *v* 39.0°C to 41.0°C), discomfort scale (“normal” or “not quite normal” *v* “some distress” or “very distressed”), previous duration of fever (≤24 hours *v* >24 hours), and current antibiotic use (yes *v* no).

### Intervention

Parents were given standardised verbal and written advice on the appropriate use of loose clothing and encouraging children to take cool fluids. The intervention was the provision of, and advice to give, the study drugs for up to 48 hours: paracetamol every 4-6 hours (maximum of four doses in 24 hours) and ibuprofen every 6-8 hours (maximum of three doses in 24 hours). Parents, research nurses, and investigators were blinded to treatment allocation by the use of identically matched placebo drugs. All parents received two medicine bottles; either both active or one containing the active drug and the other placebo. Given the differences in dosing, the parents were aware of which was paracetamol/placebo and which was ibuprofen/placebo. All liquid suspensions were sugar-free and supplied in licensed containers with child resistant caps. The dose of drug was determined by the child’s weight: paracetamol 15 mg/kg per dose and ibuprofen 10 mg/kg per dose. At the baseline visit and before randomisation the research nurse weighed the child, undressed to one layer, using scales approved for use in children (Seca, UK). Randomisation was abandoned if weight could not be established and administration of the study drug was deemed unsafe. The research nurse initially calculated the volume of suspension per dose (to the nearest 0.5 ml), which was confirmed during randomisation. The bottles of active drug contained the standard concentrations: 120 mg of paracetamol per 5 ml and 100 mg of ibuprofen per 5 ml.

The first doses were given in the presence of the research nurse and were timed to coincide with the child’s next due dose of drug—that is, at least four hours after the last dose of paracetamol and six hours after that of ibuprofen, and were never such that the maximum number of doses over a 24 hour period was exceeded. The order in which the first drug was given was determined randomly. We recorded the time that the drug was swallowed and designated that as time zero. The first four hours, after children were observed to be given the drugs and before any further drug was given, was regarded as the “efficacy period.” We asked the parents to give the drugs regularly from four to 24 hours (“proactive period”). Figure 1 describes the intervention period for the first 24 hours. We asked parents to give the drugs between 24 and 48 hours in response to their child’s symptoms (“reactive period”). At 48 hours we retrieved the study drugs and advised the parents to use over the counter preparations as required until day 5.

**Figure fig1:**
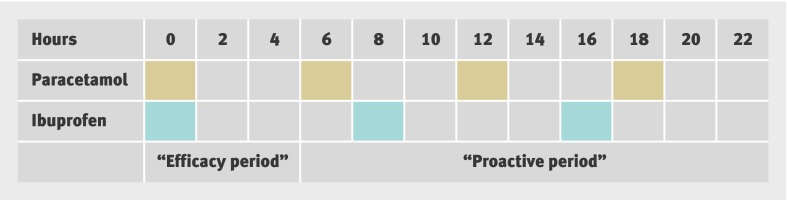
**Fig 1** Use of study drugs during first 24 hours. Shaded areas represent time that drug was to be given

### Outcomes

We timed all outcomes in relation to the administration of the first drug doses. The primary outcomes were the number of minutes without fever (<37.2°C) in the first four hours and the proportion of children reported as being normal on the discomfort scale at 48 hours. Secondary outcomes were collected at three time points. In the first 24 hours we recorded the time to temperature first falling below 37.2°C (fever clearance), the time spent without fever over 24 hours, and the proportion of children without fever associated symptoms: discomfort, reduced activity, reduced appetite, and disturbed sleep. At 48 hours and day 5 we obtained data on fever associated symptoms and temperature measured by parents. At all time points we asked parents about adverse effects.

We measured time without fever using a technique similar to that in a previous study.^20^ Using a data logger (OM-CP-RTDTEMP110; Omega Engineering, Stamford, CT) connected to an axillary temperature probe, we measured and recorded temperature every 30 seconds. Parents were asked to help their child keep the logger on for 24 hours. With support from research nurses the parents completed symptom diaries on discomfort, sleep, appetite, and activity using ordered categorical scales. Parents were asked to enter the value best representing their child’s state at the time of recording or in the previous 10 minutes. They also recorded adverse effects (defined as new symptoms or worsening of pre-existing symptoms^21^) and temperature, which they measured with a standard digital axillary thermometer.

### Sample size

In the original protocol the target difference for the time spent without fever in the first four hours was 30 minutes (with an estimated standard deviation of 80 minutes^20^) and that for the binary outcome of being rated normal on the discomfort scale at 48 hours was 60% compared with 75% (equivalent to an odds ratio of 2.0). To detect the latter comparison with 90% power and a two sided α of 0.027 (allowing for multiple comparisons between the combined therapy group and each of the two single therapy groups^22^) we required a total sample size of 747 children. Difficulties with recruitment led to the addition of the remote and community methods and a reduced achievable sample size. For time without fever we estimated a revised standard deviation of 50 minutes on the basis of the first 50 children (independent of allocation group). Along with a revised 80% power, we determined that a total sample size of 180 would allow the detection of the original target difference of 30 minutes. Sensitivity to differences in discomfort was, however, reduced, with odds ratios of only 4 or more being detectable.

### Statistical analyses

We obtained descriptive statistics to characterise children, assess baseline comparability, and compare side effects. Comparative analyses were done in Stata 9 on an intention to treat basis using linear or logistic regression and adjusting for minimisation variables. Primary comparisons were between paracetamol plus ibuprofen and either drug alone, and secondary comparisons were between paracetamol and ibuprofen, using Dunnett’s and Tukey’s adjustments, respectively, for multiple comparisons.^22^ For all “time without fever” analyses we regarded as valid only biologically plausible temperatures of more than 33°C and less than 45°C. In regression models we used the proportion of valid time under the fever threshold (with results converted into minutes or hours for presentational purposes) and we weighted these according to the amount of valid data. Secondary analyses included additional adjustment for factors showing possible imbalance at baseline and preplanned exploratory analyses for differential effects of paracetamol plus ibuprofen compared with paracetamol alone or ibuprofen alone for baseline age, temperature, discomfort, antibiotic use, and presence of otitis media. We selected otitis media because affected children might experience enhanced effects for both fever and pain.

## Results

Thirty five primary care sites in Bristol agreed to take part in the trial: NHS Direct, one walk-in centre, 30 general practices, two general practitioner out of hours cooperatives, and the emergency department of the Bristol Royal Hospital for Children. Figure 2 shows the numbers of children recruited through the three different methods. Overall, 4515 contacts were made, of which 3477 children were ineligible, most commonly (89%) because of insufficient fever. The remaining 1038 children were potentially eligible, but the temperature criterion before randomisation could not be verified in 882 because the parents did not want to commit to the study or had concerns about the drugs (669 declined) or the parents saw a clinician but left without contacting the study team (213 missed). No parent declined at randomisation, and attrition was minimal. Deviations from the protocol occurred; in the first 24 hours (23 hours and 40 minutes), 13 (7) children received an erroneous fifth dose of paracetamol and similarly 18 (13) children an erroneous fourth dose of ibuprofen. In four children, clinicians and parents but not research staff were unblinded to treatment allocation.

**Figure fig2:**
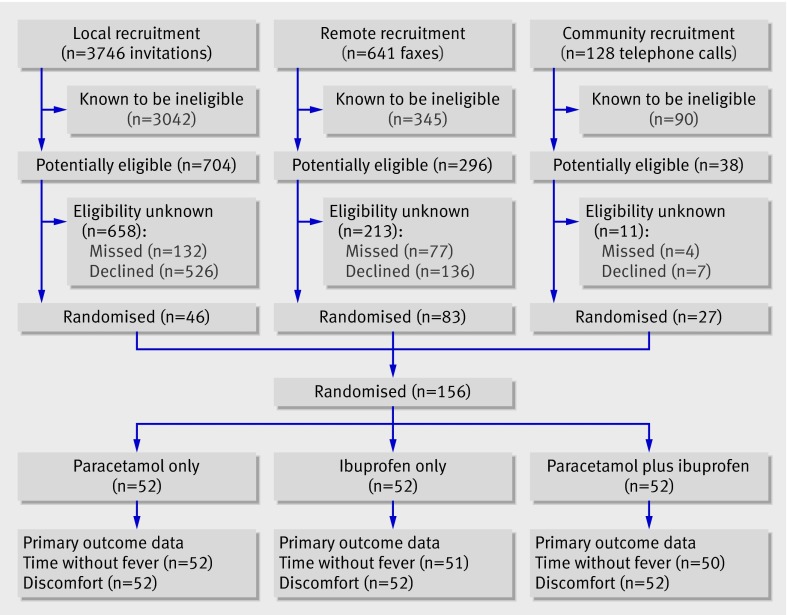
**Fig 2** Participant flow through trial

### Descriptive results

The groups were comparable at baseline, although potentially influential differences existed for sex, method of recruitment, and activity (table 1). Since additional adjustment for these variables had negligible effects in all analyses only minimisation variables were adjusted for in the comparative analyses. Nearly all the children were unwell, with more than 90% experiencing discomfort, reduced activity, abnormal appetite, or abnormal sleep (table 1).

**Table 1 tbl1:** Baseline characteristics of children with fever randomised to three treatment groups. Values are numbers (percentages) of children unless stated otherwise

Characteristic	Paracetamol (n=52)	Ibuprofen (n=52)	Paracetamol plus ibuprofen (n=52)
Boy	26 (50)	37 (71)	25 (48)
Girl	26 (50)	15 (29)	27 (52)
Mean (SD) weight (kg)	13.0 (4.2)	13.4 (3.9)	12.6 (3.3)
Mean (SD) age (months)	28.7 (17.7)	28.1 (17.4)	25.1 (13.4)
Age (months)*:			
6-17	20 (38)	18 (35)	19 (37)
18-71	32 (62)	34 (65)	33 (63)
Mean (SD) baseline temperature (°C)	38.6 (0.6)	38.6 (0.6)	38.6 (0.6)
Temperature (°C)*:			
<39	37 (71)	37 (71)	39 (75)
39-41	15 (29)	15 (29)	13 (25)
Fever duration (hours)*:			
≤24	18 (35)	19 (37)	19 (37)
>24	34 (65)	33 (63)	33 (63)
Antibiotic use*:			
Yes	14 (27)	15 (29)	17 (33)
No	38 (73)	37 (71)	35 (67)
Paracetamol use 4-6 hours before randomisation:			
Yes	20 (38)	17 (33)	20 (38)
No	32 (62)	35 (67)	32 (62)
Ibuprofen use 6-8 hours before randomisation:			
Yes	4 (8)	2 (4)	3 (6)
No	48 (92)	50 (96)	49 (94)
Discomfort*:			
Normal	3 (6)	5 (9)	5 (9)
Not quite normal	31 (60)	27 (52)	30 (58)
Some pain or distress	18 (34)	18 (35)	14 (27)
Crying or very distressed	0 (0)	2 (4)	3 (6)
Activity:			
Normal	3 (6)	4 (8)	4 (8)
Quiet for longer than usual	12 (23)	18 (35)	23 (45)
Hardly moving about	31 (60)	19 (36)	19 (36)
Not moving about willingly	6 (11)	11 (21)	6 (11)
Appetite:			
Normal	5 (10)	3 (6)	4 (8)
Eating less than normal	12 (23)	14 (27)	10 (19)
Eating much less than normal	35 (67)	33 (63)	36 (69)
Vomiting or refusing food or drink	0 (0)	2 (4)	2 (4)
Sleep:			
Normal	8 (15)	3 (6)	4 (8)
More than usual	20 (38)	21 (40)	20 (38)
More disturbed than usual	9 (17)	15 (29)	10 (19)
A lot more disturbed than usual	15 (29)	13 (25)	18 (35)
Recruitment method:			
Local	17 (33)	18 (35)	10 (19)
Remote	27 (52)	26 (50)	31 (60)
Community	8 (15)	8 (15)	11 (21)
Ethnicity:			
White	47 (90)	47 (90)	44 (85)
Other	5 (10)	5 (10)	8 (15)
Diagnosis:			
Otitis media	7 (14)	11 (20)	8 (14)
Respiratory tract infection	12 (23)	15 (28)	17 (33)
Non-specific viral illness	21 (40)	20 (37)	16 (31)
Other	12 (23)	8 (15)	11 (22)
Previous febrile convulsion:			
Yes	2 (4)	1 (2)	2 (4)
No	50 (96)	51(9)	50 (96)
Asthma:			
Yes	9 (17)	4 (8)	6 (12)
No	43 (83)	48 (92)	46 (88)

*Minimisation criterion (baseline temperature included as continuous variable in all models; baseline discomfort minimised distinguishing the top and bottom two levels because of anticipated frequencies but included as four level categorical variable in all models).

The median time between randomisation and giving the first dose of study drug was eight minutes for paracetamol plus ibuprofen and nine minutes for paracetamol and for ibuprofen. The mean number of valid minutes for time without fever (temperature >33°C and <45°C) in the first four hours (240 minutes) was 219 for children receiving paracetamol, 211 for ibuprofen, and 202 for paracetamol plus ibuprofen. The respective times over 24 hours (1440 minutes) were 1078, 1029, and 1051 minutes. For time without fever in the first four valid hours (and the corresponding secondary outcome within 24 valid hours), children receiving paracetamol plus ibuprofen spent more time without fever than those given ibuprofen and, in turn, those given paracetamol (table 2). Fever clearance was faster in children given paracetamol plus ibuprofen than in those given paracetamol but was similar for those given ibuprofen. Children given paracetamol plus ibuprofen spent less time with fever over 24 hours than those given either drug alone. A suggestion was that more fever associated symptoms had normalised in children given ibuprofen than the other treatments at 24 and 48 hours, but by day 5 these trends had largely disappeared.

**Table 2 tbl2:** Descriptive statistics of outcomes (time without fever and no discomfort) at selected times. Values are numbers (percentages) unless stated otherwise

Outcomes	Paracetamol (n=52)	Ibuprofen (n=52)	Paracetamol plus ibuprofen (n=52)
**Primary outcomes**			
Mean (SD) time without fever in first 4 hours (minutes)*	116.2 (65.0)	156.0 (57.6)	171.1 (40.8)
No discomfort at 48 hours†	34 (65)	37 (71)	36 (69)
**Secondary outcomes:**			
Outcomes at 24 hours:			
Mean (SD) time until first fever clearance (minutes)‡	71.0 (69.1)	42.2 (33.5)	45.5 (34.3)
Mean (SD) time without fever in first 24 hours (minutes)*	940.3 (362.9)	1055.2 (329.7)	1217.4 (237.6)
No discomfort†	22 (44)	36 (69)	29 (56)
Normal activity†	20 (40)	20 (58)	23 (48)
Normal appetite†	10 (21)	14 (27)	14 (29)
Normal sleep†	17 (37)	13 (50)	20 (37)
Outcomes at 48 hours:			
Mean (SD) temperature (°C)§	36.4 (0.89)	36.4 (0.85)	36.6 (1.01)
Normal activity†	31 (60)	37 (73)	28 (54)
Normal appetite†	21 (41)	22 (44)	21(41)
Normal sleep†	27 (52)	31 (61)	25 (48)
Outcomes at day 5:			
Mean (SD) temperature (°C)**	36.2 (0.93)	36.1 (0.78)	36.0 (0.66)
No discomfort†	43 (88)	38 (81)	38 (76)
Normal activity†	44 (90)	39 (85)	37 (73)
Normal appetite†	29 (58)	29 (59)	32 (62)
Normal sleep†	31 (62)	25 (50)	27 (53)

*Time spent with temperature less than 37.2°C in first four hours after first dose of drug, using number of valid 30 second interval points from data logger; unknown for zero, one, and two children in three groups, respectively, by four hours, and zero, two, and two, respectively, by 24 hours. Time without fever over first four hours was 48 minutes for paracetamol, 65 minutes for ibuprofen, and 71 minutes for paracetamol plus ibuprofen and for time without fever in first 24 hours was 65 minutes for paracetamol, 73 minutes for ibuprofen, and 84 minutes for paracetamol plus ibuprofen.

†Children reported at relevant time to be “normal” (see table 1); denominators may vary owing to missing data (in most cases fewer than four children).

‡Time from baseline until temperature first falls below 37.2°C; unknown for five children (zero, two, and three, respectively) and right censored at 240 minutes for three children.

§Measured by research nurse; unknown for one, five, and two children, respectively.

**Measured by parent; unknown for four, seven, and three children, respectively.

### Comparative analyses

#### Primary outcomes

Strong evidence was found of more time spent without fever in the first four hours among children given paracetamol plus ibuprofen than those given paracetamol, and likewise for children given ibuprofen than those given paracetamol (table 3). Moreover, both point estimates exceeded the 30 minute target difference, as did the lower confidence limit for the primary comparison. The confidence interval and P value suggest little difference between giving paracetamol plus ibuprofen and giving ibuprofen alone.

The low power for fever associated discomfort at 48 hours was reflected by the large P values and wide confidence intervals for all three comparisons, although the largest point estimate and upper confidence limit favoured ibuprofen over paracetamol. The lowest P value from subgroup analyses for the primary outcomes was 0.14.

**Table 3 tbl3:** Regression models for time without fever over first four hours (240 minutes) and no discomfort at 48 hours, adjusting for minimisation

Outcomes	Primary comparisons		Secondary comparison: ibuprofen *v* paracetamol
	Paracetamol plus ibuprofen *v* paracetamol	Paracetamol plus ibuprofen *v* ibuprofen	
Time without fever in first 4 hours*:			
Adjusted difference (minutes)	55.3	16.2	39.0
95% confidence interval	33.1 to 77.5†	−7.0 to 39.4†	15.9 to 61.0‡
P value	<0.001†	0.2†	<0.001‡
No discomfort at 48 hours§:			
Adjusted odds ratio	1.33	0.89	1.50
95% confidence interval	0.49 to 3.56†	0.32 to 2.43†	0.53 to 4.26‡
P value	0.7†	>0.8†	>0.5‡

*Weighted by number of time points in first four hours contributing valid data on temperature. Positive differences indicate additional minutes below 37.2°C for first named treatment group compared with comparator.

†Primary comparisons after applying Dunnett’s correction (approximate P values obtained using extrapolation from limited published values^21^; uncorrected P values were <0.001 and 0.11 for time without fever, 0.53 and 0.79 for discomfort).

‡Secondary comparison after applying Tukey’s correction (P values obtained using interpolation from extensive published values^21^; <0.001 for temperature, 0.37 for discomfort).

§Odds of being well compared with not being well.

#### Secondary outcomes

The comparison of fever clearance was consistent with the primary outcome for time without fever: strong evidence suggested that paracetamol plus ibuprofen had a faster effect than paracetamol alone, and ibuprofen alone had a faster effect than paracetamol alone (table 4). Giving paracetamol plus ibuprofen over 24 hours increased time without fever by 4.4 hours compared with paracetamol and by 2.5 hours compared with ibuprofen.

No consistent evidence of effect for fever associated symptoms from 24 hours to day 5 was seen, but odds ratios tended to favour ibuprofen more than the other treatments at 24 and 48 hours (data not shown).

**Table 4 tbl4:** Regression models for time without fever up to 24 hours, adjusting for minimisation

Outcomes	Primary comparisons		Secondary comparison: ibuprofen *v* paracetamol
	Paracetamol plus ibuprofen *v* paracetamol	Paracetamol plus ibuprofen *v* ibuprofen	
Time until first fever clearance*:			
Adjusted difference (minutes)	−23.5	3.0	−26.3
95% confidence interval	−44.8 to −2.2†	−18.3 to 24.4†	−48.3 to −4.3‡
P value	0.025†	>0.8†	0.015‡
Time without fever in first 24 hours§:			
Adjusted difference (hours)	4.4	2.5	1.9
95% confidence interval	2.4 to 6.3†	0.6 to 4.4†	−0.2 to 4.0‡
P value	<0.001†	0.008†	0.076‡

*Negative differences indicate that first named treatment group has faster fever clearance time than comparator group.

†Primary comparisons after applying Dunnett’s correction (uncorrected P values were 0.016 and 0.75 for fever clearance, <0.001 and 0.005 for time without fever).

‡Secondary comparison after applying Tukey’s correction (uncorrected P values were 0.006 for fever clearance, 0.033 for time without fever).

§Weighted by number of time points in first 24 hours contributing valid data on temperature; positive differences indicate additional hours with temperature less than 37.2°C for first named treatment group than for comparator.

### Mean temperature by treatment group

Figure 3 shows the mean temperature every 15 minutes by treatment group with the proportion of children febrile at corresponding two hourly time points. Ibuprofen and paracetamol plus ibuprofen reduced children’s temperatures faster and for longer than paracetamol in the first four hours, and paracetamol plus ibuprofen was superior to either drug alone in reducing mean temperatures over 24 hours. A rise in mean temperature was seen for children in the ibuprofen group, which then fell just after six hours, coinciding with the earliest time that parents were advised that a second dose of ibuprofen could be given. This rise may have been prevented in the other groups by paracetamol, which could have been given at four hours.

The mean temperatures in the graph are lower than might be expected biologically. This could be explained by the choice of axillary thermometry, which is known to record temperatures around 0.8°C lower than rectal digital thermometers,^23^ or by the liberal definition of valid temperature used in this study, or both. A sensitivity analysis excluding temperatures below 35°C raised the mean temperatures but not the relative positions of the group means.

**Figure fig3:**
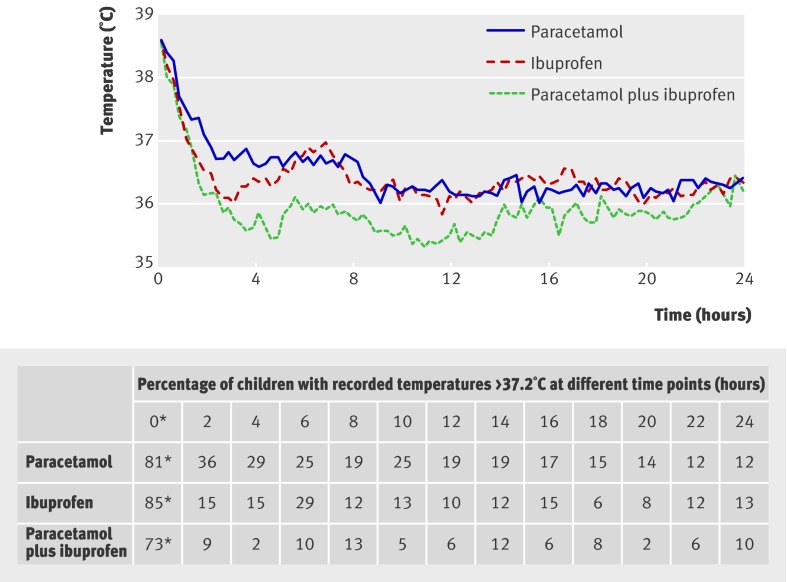
**Fig 3** Mean temperature over first 24 hours after randomisation, by treatment group. *All children had temperatures greater than 37.2°C at baseline eligibility assessment, as measured by standard digital axillary thermometry. Temperature measured using a data logger was less than 37.2°C for 19 children because of delays between digital thermometry measure and drug dosing and differences between digital and data logger thermometry methods

### Relation between discomfort and temperature

Given the low power for treatment effects on discomfort, a repeated measure analysis was used to explore the relation between all discomfort measures recorded across up to eight time points to 48 hours and their coinciding mean digital axillary thermometer measures. The mean temperatures were 36.4°C for children who scored normal on the discomfort scale, 37.2°C for those who scored not quite normal, 38.1°C for those who scored some pain or distress, and 38.3°C for those who scored crying or very distressed.

### Adverse effects

The most common adverse effects were diarrhoea and vomiting, which were equally distributed between groups (table 5). The overall number of children experiencing adverse events was, however, too small to make meaningful comparisons between treatments. Five children were admitted to hospital (constituting serious adverse events^21^): one child in the paracetamol group, three in the ibuprofen group, and one in the paracetamol plus ibuprofen group. On independent review none was considered to be related to the study process or drugs.

**Table 5 tbl5:** Five most common adverse effects. Values are numbers of children

Adverse effect	Paracetamol (n=52)	Ibuprofen (n=52)	Paracetamol plus ibuprofen (n=52)
Diarrhoea	10	9	12
Vomiting	6	3	2
Rash	2	2	1
Cough	2	0	1
Cold to touch	0	3	2

### Dosing of study drugs

All 52 children in each of the three groups were given, as per protocol, their first dose of study drug under nurse supervision (table 6). The recommended maximum four doses of paracetamol or placebo in the first 24 hours was received by 65% of children given paracetamol, 46% given ibuprofen, and 42% given paracetamol plus ibuprofen, with this recommended maximum exceeded by 12%, 6%, and 8%, respectively. The corresponding percentages receiving the recommended maximum three doses of ibuprofen or placebo in 24 hours were 73%, 75%, and 71% and those exceeding this recommended maximum were 13%, 12%, and 13%. All percentages were much lower at 48 hours.

**Table 6 tbl6:** Number of doses of paracetamol alone or ibuprofen alone over 24 and 48 hours. Values are numbers (percentages) of children

Drug use (dose No)	Paracetamol (n=52)	Ibuprofen (n=52)	Paracetamol plus ibuprofen (n=52)
Paracetamol or placebo in 24 hours:			
1	52 (100)	52 (100)	52 (100)
2	52 (100)	49 (94)	51 (98)
3	48 (92)	44 (85)	47 (90)
4	34 (65)	24 (46)	22 (42)
5	6 (12)	3 (6)	4 (8)
Paracetamol or placebo in 48 hours:			
1	52 (100)	52 (100)	52 (100)
2	52 (100)	49 (94)	51 (98)
3	50 (96)	49 (94)	49 (94)
4	42 (81)	39 (75)	38 (73)
5	35 (67)	26 (50)	24 (46)
6	20 (38)	11 (21)	15 (29)
7	8 (15)	6 (12)	6 (12)
8	3 (6)	1 (2)	1 (2)
9	—	1 (2)	—
Ibuprofen or placebo in 24 hours:			
1	52 (100)	52 (100)	52 (100)
2	51 (98)	48 (92)	51 (98)
3	38 (73)	39 (75)	37 (71)
4	7 (13)	6 (12)	7 (13)
5	—	—	2 (4)
Ibuprofen or placebo in 48 hours:			
1	52 (100)	52 (100)	52 (100)
2	51 (98)	49 (94)	51 (98)
3	45 (87)	45 (87)	46 (88)
4	32 (62)	34 (65)	29 (56)
5	18 (35)	5 (10)	18 (35)
6	7 (13)	4 (8)	10 (19)
7	—	1 (2)	3 (6)

### Blinding

The success of blinding was assessed at the nurse’s visit at 48 hours, when parents were asked to guess treatment allocation. Taking “I don’t know” responses to either drug as failure to guess correctly, allocation was guessed correctly by 16 (31%) parents in the paracetamol group, 17 (33%) in the ibuprofen group, and 9 (17%) in the paracetamol plus ibuprofen group, compared with the 33% expected by chance. Excluding “I don’t know” responses increased these percentages to 50% (32 parents), 53% (n=32), and 43% (n=21), respectively.

## Discussion

In febrile children we found strong evidence of faster time to fever clearance and more prolonged time without fever in the first four hours favouring the use of paracetamol plus ibuprofen and ibuprofen over paracetamol, but no evidence of any difference between paracetamol plus ibuprofen and ibuprofen alone. In the first 24 hours strong evidence suggested more time without fever favouring paracetamol plus ibuprofen over either drug alone. We found no evidence of differences in fever associated discomfort at 48 hours. The frequency of adverse effects did not seem to differ between groups.

### Comparison with existing literature

Using continuous thermometry we compared the effects of two antipyretics combined with either drug alone using maximum licensed, repeated doses in children recruited from and managed in the community. Previous studies have recruited from secondary care,^11^ ^12^ ^14^ ^15^ investigated the effects of single doses,^12^ ^14^ and did not use continuous thermometry. The finding that ibuprofen was found to be more effective than paracetamol in the first four hours is consistent with the literature.^9^

### Strengths and limitations of the study

The study has four main strengths. Firstly, its internal validity: randomisation was concealed, nurses and investigators were blinded to allocation, and attrition was minimal. Secondly, the intervention and follow-up periods were long enough to enable a fair comparison between multiple doses of antipyretics with differing times to maximum effect.^16^ Thirdly, we used continuous thermometry to generate the objective and intuitive outcome of time without fever. Finally, we recruited and followed up children in the community, where most cases of fever are managed.

We are aware of five possible weaknesses of the study. Firstly, because we had no placebo only group our data cannot inform the decision on whether to use antipyretics. This was a deliberate design decision as we thought that parents would not have participated if there had been a placebo only group. This judgment is supported by the fact that over 80% of parents in the study said that they would not have participated in such a trial. Three previous trials have, however, shown that paracetamol and ibuprofen given separately are more effective than placebo,^6^ ^7^ ^8^ and one trial found that paracetamol is more effective at relieving fever than unwrapping children.^20^

Secondly, the recruited sample did not give sufficient power to detect plausible differences in discomfort. This is disappointing, given the importance of this question to the public and research community. Other research has, however, suggested that the use of two drugs combined compared with one alone does confer additional benefit on symptoms^13^ and we did find a relation between increasing discomfort and worsening fever, suggesting that with adequate power the effects on symptoms might have followed those of temperature.

Thirdly, an axillary temperature of 37.8°C might not be regarded as denoting fever. Since no agreed definition of fever or how to measure temperature exists,^24^ to a limited extent its selection was arbitrary. For example, disagreement between thermometer types and measurement sites means this could represent a rectal temperature of as much as 39.7°C.^23^ Temperature is such a dynamic variable that although many children did not meet our criterion for temperature before randomisation, most were already being treated for a febrile illness and their parents and doctors thought that treatment with up to two drugs was warranted. The mean temperature at baseline was 38.5°C (table 1), a temperature at which 90% of doctors and 70% of nurses would recommend treatment,^25^ and most of the children were unwell with febrile illness as it affected their comfort, appetite, activity, and sleep.

Fourthly, the success of blinding was assessed at the 48 hour nurse visit by asking parents to guess which drugs were active. Overall, the 153 parents who responded were not able to guess treatment, but the 83 who expressed a definite opinion did identify allocation more often than would be expected by chance. Although we carried out blinded taste tests and volunteers could not distinguish placebo from active drugs, some parents may have been better able to do so because they had more time to compare study drugs with known products in the home as well as observing their children’s responses to treatment. Although this could have influenced the parental recording of the discomfort outcome, we do not see how it could influence the outcome of time without fever.

Finally, given the challenges of recruitment, our sample might not be representative of the general population. For example, we do not know if the possibility of receiving either or both drugs combined and the severity of the child’s illness influenced parents’ decisions to participate. If this was the case, we think these factors are more likely to be associated with differences in parental attitudes to illness than the children’s response to the drugs. The most common reason for ineligibility was insufficient fever, a factor we think is unlikely to be associated with any other physiological marker of response to drugs.

### Implications of this research

It is good practice for parents, nurses, and doctors who have made the decision to treat young, unwell children with fever, to use the minimum number of drugs possible.^5^ Although other studies have shown that paracetamol is superior to placebo,^6^ ^7^ ^8^ our study suggests that those wanting to achieve faster and more prolonged fever relief in the first four hours should use ibuprofen in preference to paracetamol. Similarly, where symptoms are expected to last at least 24 hours (probably most children with more severe symptoms at the onset of illness), those wanting to maximise the time without fever should probably start with ibuprofen but also consider paracetamol plus ibuprofen. Pragmatically, although our trial design did not specifically address this, we speculate that if a child remains unwell after a first dose of ibuprofen, subsequent alternation of paracetamol and ibuprofen for 24 hours would be more effective than either drug alone. This speculation is supported by a recent study showing that paracetamol was more effective than placebo when added to ibuprofen.^14^ The decision to start with ibuprofen or paracetamol plus ibuprofen, however, should also be influenced by an assessment of the benefits (an additional 2.5 hours without fever) compared with the risk of unintentionally exceeding the maximum recommended dose owing to the additional complexity of using two drugs. This risk is not theoretical. Even in the context of this supervised trial, between 6% and 13% of parents exceeded the maximum number of recommended doses in the first 24 hours.

The pragmatism of the intervention changed with time, moving from efficacy in the first four hours to effectiveness in the second 24 hours. By 48 hours, considerably fewer study drugs were being given and this could partly explain the observed lack of effects on discomfort at this time. In the community, paracetamol and ibuprofen are usually dosed by age, and we recognise that calculating doses by weight means the results may inform primary and secondary care practice more than practice at home. We decided against a dose by age regimen, however, for two reasons. Firstly, given the recommendation of the children’s national service framework to dose by weight^26^ and the dose by weight presentations in the British national formulary for children,^18^ we believe that in the future more medicines for children will be given by weight. Secondly, we wanted to ensure that heavier children for their age received a therapeutic dose and to avoid exceeding the normal recommended dose for children who were light for their age. Comparing dose by weight with dose by age shows that children can receive as much as 50% more^27^ or 50% less paracetamol and 100% more ibuprofen.

Medicine bottles in the United States contain dosing advice by both age and weight and although healthcare professionals can clearly calculate dose by weight, we think two steps are needed before parents can routinely use weight to determine dose in other countries. Firstly, studies should investigate the safety implications of any differences between estimates of children’s weights measured by parents using domestic scales (or recently recorded weights in parent held children’s health records) and those measured by professionals using paediatric scales. Secondly, suppliers of antipyretics could consider routinely including dose by weight tables. Given that the complexity of using two drugs over a 24 hour period is more likely to lead to inadvertently exceeding the maximum recommended dose, we also believe that multiple blank charts should be supplied for parents to record when medicines have been given and how much.

Recent case reports have highlighted the concern about renal toxicity in dehydrated children given ibuprofen.^28^ ^29^ Although this serious effect is rare, we excluded children with dehydration from our trial and believe that ibuprofen should not routinely be given to children with, or at risk of, dehydration. Good evidence shows, however, that ibuprofen is as safe as paracetamol for children with asthma, where there is no evidence of sensitivity to non-steroidal anti-inflammatory drugs.^30^

We agree with the guidelines for fever from the National Institute for Health and Clinical Excellence (NICE) that antipyretics should be used only when children have fever associated with other symptoms,^5^ although further research is needed to establish the effectiveness of antipyretics for the relief of these symptoms. However, we believe that the guidance on the use of two drugs combined need not be so cautious now that there is good evidence of superiority for both drugs over one drug for increasing time without fever over 24 hours.

### Conclusion

Doctors, nurses, pharmacists, and parents wanting to use medicines to treat young, unwell children with fever should be advised to use ibuprofen first and to consider the relative benefits and risks of using paracetamol plus ibuprofen over a 24 hour period. There is no evidence from the accompanying cost effectiveness evaluation to contradict these findings.^17^

**What is already known on this topic**

Paracetamol plus ibuprofen are being increasingly used at home and in primary and secondary care for the relief of fever and its associated symptoms
Five previous trials of combined therapy mostly tested single doses for children in secondary care and reached conflicting conclusions

**What this study adds**

In the first four hours, temperature is reduced faster and for longer in children given ibuprofen than in those given paracetamol
In the first 24 hours, children given both drugs spent 4.4 hours less time with fever than those given paracetamol and 2.5 hours less time with fever than those given ibuprofen.
Parents and healthcare professionals should consider ibuprofen first and the relative benefits and risks of using combined therapy over 24 hours

## Notes

We thank Avon, Gloucestershire, and Wiltshire NHS Direct; the Bristol general practitioner practices; the south Bristol walk-in centre; the emergency department of the Bristol Royal Hospital for Children; the children and parents who participated; the South West Medicines for Children Local Research Network; the research nurse team W Horseman, J Farrimond, R Powell, S Shatford, P Richards; the South West Medicines for Children Local Research Network nurse V Payne; W Patterson (trial coordinator); S Doohan and S Burke (project administrators); K Schroeder, M Weiss, and A Emond (co-applicants); Sara Whitburn (proof and background reading); K Pitcher (data entry and quality); the trial steering committee (AL Kinmonth, C Butler, J Peacock, M Blythe, and P Denyer); and the data monitoring and safety committee (R Bragonier, S Kerry, and J Chudleigh).

Ethical approval: Bath research ethics committee (reference No 04/Q2001/197).

Provenance and peer review: Not commissioned; externally peer reviewed.

## Notes

**Cite this as:** *BMJ* 2008;337:a1302
